# Congruency of multimodal data-driven personalization with shared decision-making for StayFine: individualized app-based relapse prevention for anxiety and depression in young people

**DOI:** 10.3389/fpsyt.2023.1229713

**Published:** 2023-09-29

**Authors:** Bas E. A. M. Kooiman, Suzanne J. Robberegt, Casper J. Albers, Claudi L. H. Bockting, Yvonne A. J. Stikkelbroek, Maaike H. Nauta

**Affiliations:** ^1^Department of Clinical Psychology and Experimental Psychopathology, Faculty of Behavioural and Social Sciences, University of Groningen, Groningen, Netherlands; ^2^Depression Expertise Centre-Youth, GGZ Oost Brabant, Boekel, Netherlands; ^3^Department of Psychiatry, Amsterdam University Medical Centres–Location AMC, Amsterdam Public Health, University of Amsterdam, Amsterdam, Netherlands; ^4^Department of Psychometrics and Statistics, Faculty of Behavioural and Social Sciences, University of Groningen, Groningen, Netherlands; ^5^Centre for Urban Mental Health, University of Amsterdam, Amsterdam, Netherlands; ^6^Department of Clinical Child and Family Studies, Faculty of Social and Behavioural Sciences, Utrecht University, Utrecht, Netherlands; ^7^Accare Child Study Centre, Groningen, Netherlands

**Keywords:** personalization, relapse prevention, network analysis, expert by experience, depression and mood disorders, anxiety disorders, child and adolescent psychiatry, shared decision-making

## Abstract

Tailoring interventions to the individual has been hypothesized to improve treatment efficacy. Personalization of target-specific underlying mechanisms might improve treatment effects as well as adherence. Data-driven personalization of treatment, however, is still in its infancy, especially concerning the integration of multiple sources of data-driven advice with shared decision-making. This study describes an innovative type of data-driven personalization in the context of StayFine, a guided app-based relapse prevention intervention for 13- to 21-year-olds in remission of anxiety or depressive disorders (*n* = 74). Participants receive six modules, of which three are chosen from five optional modules. Optional modules are *Enhancing Positive Affect, Behavioral Activation, Exposure, Sleep*, and *Wellness*. All participants receive *Psycho-Education, Cognitive Restructuring*, and a *Relapse Prevention Plan*. The personalization approach is based on four sources: (1) prior diagnoses (diagnostic interview), (2) transdiagnostic psychological factors (online self-report questionnaires), (3) individual symptom networks (ecological momentary assessment, based on a two-week diary with six time points per day), and subsequently, (4) patient preference based on shared decision-making with a trained expert by experience. This study details and evaluates this innovative type of personalization approach, comparing the congruency of advised modules between the data-driven sources (1–3) with one another and with the chosen modules during the shared decision-making process (4). The results show that sources of data-driven personalization provide complementary advice rather than a confirmatory one. The indications of the modules *Exposure* and *Behavioral Activation* were mostly based on the diagnostic interview, *Sleep* on the questionnaires, and *Enhancing Positive Affect* on the network model. Shared decision-making showed a preference for modules improving positive concepts rather than combating negative ones, as an addition to the data-driven advice. Future studies need to test whether treatment outcomes and dropout rates are improved through personalization.

## 1. Introduction

Adolescents and young adults with anxiety and depressive disorders have not only impairments in functioning with a high burden of disease, but those in remission are also prone to relapse (39–72% over 12–15 years) (1, 2). How to optimally prevent relapse in youth remains a question; however, since there is mild to no depression or anxiety, randomized controlled trials have been conducted to study relapse prevention in youth (3). For psychological treatments in general, standardized evidence-based treatments (EBTs) outperform usual care (ES = 0.25) (4) and are positively regarded and fairly well-used (5). Yet, they possess several limitations regarding efficacy (achieving desired results), efficiency (reusability and modification potential), and effectiveness (generalizability and feasibility) (6). Regarding efficacy, using cognitive behavioral therapy (CBT) as an example, there seem to be ceilings in reaching remission rates for anxiety (46.8–49.4%) (7) and depression (38–82%) (8) in youth. Possibly because standardized EBTs, as opposed to person-specific approaches, assume interventions can target homogenous clinical symptom profiles between individuals (9). Concerning effectiveness and efficiency, practitioners have mentioned that standardized EBTs have difficulty handling more complex cases, leave little room to individualize in their strict and uniform structure, and hamper spontaneity and flexibility during treatment (10). This can lead to dropout in youth with more severe and enduring mental health problems through demotivation or iatrogenic harm, which may create pessimism for and in future treatment (11). Thus, an optimal balance between efficacious EBT ingredients and effective and efficient tailoring of treatment to the individual profile of a patient seems warranted (12).

Personalized interventions concern treatments tailored to the individual through assessment of pivotal individual characteristics, adjusting the treatment to the individual based on these characteristics (12, 13). Examples of personalization methods are subgroup adaptation, measurement feedback systems, individualized metrics, predictive analytics, and modularity (13, 14). Generally, characteristics upon which to personalize are determined through the findings of prior studies, meta-analyses, and meta-reviews identifying treatment moderators. These are baseline or pre-randomization characteristics that interact with the treatment condition to affect treatment efficacy, thereby informing which treatment works for whom and under what circumstances (15). Similar is the creation of a matching factor for treatment allocation based on patient profile (16), such as the Probability of Treatment Benefit and the Personalized Advantage Index (17, 18). An example of an adjustment factor during treatment is the Trier Treatment Navigator, in which recommendations for the lowest dropout risk and optimal treatment are adjusted during treatment based on warning signal predictors (19). The aforementioned factors contain characteristics such as baseline symptom severity, comorbid personality disorder, and prior medication (trials), as well as gender, employment status, marital status, somatic complaints, cognitive problems, paranoid symptoms, interpersonal self-sacrificing, attributional style, and (number of) life events (17, 20, 21). So far, however, identification of treatment moderators has borne less robust results (14, 22–24). Furthermore, these factors and moderators are created retrospectively through *post-hoc* simulation techniques for allocation in future studies. This assumes that participant characteristics and study treatment protocols are similar between studies, which is not necessarily so (19, 25). In this study, a prospective personalization method is used for personalization within the same study, using modularity.

Modular therapy concerns a treatment divided into smaller blocks (modules) that have partial decomposability (being meaningful and functional), proper functioning (producing the intended result), a standardized interface (structured inter-communication and connection), and the ability to handle information hiding (encapsulation) (6). As such, compared to standardized EBTs, a modular approach aims to increase efficiency and effectiveness through reusability, reorganization, and adaptability. With these qualities, modularity also strikes an optimal balance between flexible tailoring through linking modules and being able to apply known efficacious treatment ingredients within modules. While sparse, some personalized modular therapies for anxiety or depression in youth exist, overall outperforming care as usual and performing similar to standardized EBTs. One example is the Modular Approach to Therapy for Children with Anxiety, Depression, Trauma or Conduct problems (MATCH-ADTC), in which different symptoms are monitored throughout treatment, and modules aiming at anxiety, depression, posttraumatic stress disorder, or conduct problems can be chosen by the therapist, using monitored data as input for module selection (26–30).

The only studied modular personalized relapse intervention in youth is the Relapse Prevention Cognitive Behavioral Therapy (RP-CBT) against depression [Kennard et al. (31); see recent meta-analysis by Robberegt et al. (3)]. This 8–11-session sequential intervention includes—after psycho-education—modules targeting core skills tailored to the residual symptoms—including behavioral coping, negative automatic thoughts, cognitive restructuring, and problem-solving—with optionally additional skill modules—including emotional regulation, social skills, assertiveness training, and relaxation training. The tail end consists of a wellness component, the creation of a relapse prevention and wellness plan, and three optional booster sessions. All patients start with core skills, based on which residual depressive symptoms and clinical issues are prominent. In the case illustration, this was initially determined by the semi-structured diagnostic interview, the K-SADS-PL (32), and clinical issues as measured by the Children's Hassles Scale (CHS) (33). During the intervention, adjustments and additional sessions, including additional skills and the content of the booster sessions, were based on clinical decision-making with input of the patient using a created timeline. After acute phase pharmacotherapy, RP-CBT combined with antidepressant medication management had a lesser risk of relapse and non-significant dropout rate differences compared to medication management alone (31, 34).

While the first steps toward personalized relapse prevention interventions show promising results, there is room for improvement in other personalized treatment approaches (14, 22, 35). This is not surprising considering the novelty of personalization research, even though the concept has roots in the 1960s (13, 18, 36). Often, theoretical underpinnings that can improve personalization treatment efficacy remain understudied. One of these is the module selection procedure for individual treatment packages based on personalized treatment ingredients (35). Few protocols offer guidance on how to use, combine, or overrule collected data in decision-making during the selection process (37). Studying 20 modular therapy protocols, mostly anxiety and depression in youth, Venturo-Conerly and colleagues (37) found that—as with RP-CBT—module selection was mostly based on baseline assessments (95%), while decision-making was primarily put upon the primary clinician (100%), less often accompanied by patient input (40%). The absence of data or guidance could be disadvantageous as clinical judgment is not without its fallacies, such as personal preference and bias. Statistical prediction outperforms clinical prediction with a small but consistent and reliable effect (38), as it did for treatment allocation to CBT vs. counseling in depressive adults based on baseline sociodemographic and clinical predictors (39). Therefore, how to personalize treatment packages in modular treatment, especially regarding the integration of data-driven advice in clinical decision-making, is a question deserving of further examination (37, 40).

This study describes and critically evaluates the congruency of a novel multimodal data-driven personalization approach in the context of StayFine, a modular guided app-based relapse prevention intervention for 13- to 21-year-old participants in remission of anxiety and/or depressive disorders (41). During StayFine, participants complete six modules. Three are a personalized selection of five modules, with options including *Enhancing Positive Affect, Behavioral Activation, Exposure, Sleep*, and *Wellness*. The other three are fixed, starting with *Psycho-education* and subsequently *Cognitive restructuring*, and ending with creating a personal *relapse prevention plan* based on all modules. The four-step personalization approach, with each step indicating separate modules, consists of three data-driven sources with feedback rules followed by shared decision-making. The three sources are as follows: (1) prior diagnoses using a diagnostic interview, (2) transdiagnostic psychological factors using online self-report questionnaires, and (3) individual symptom networks using ecological momentary assessment (EMA). Subsequently, (4) patient preference is included through shared decision-making with a trained expert by experience. By implementing multiple modes of measuring a personalization characteristic, one potentially increases the robustness of the module selection and, therefore, the efficacy and efficiency of the personalized treatment package as a whole as each assessment type has its own benefits and disadvantages.

The first personalization step was examining the prior diagnostic classification to consider which module could address underlying negative residual mechanisms. This is similar to the case illustration of RP-CBT (31), using both a diagnostic interview and questionnaires. A diagnostic interview is often used as part of an anamnesis to determine which form of acute treatment one requires. It includes the clinically trained view of the assessor, who can evaluate answers through appended questions, repetition, or paraphrasing while the interview takes place.

Second, questionnaires were conducted to measure transdiagnostic underlying mechanisms to be targeted in specific intervention modules. Questionnaires are time-efficient and less energy-consuming for the participant compared to qualitative data gathering, at the cost of potential inter-individual differences in response distortions such as acquiescence, extremity vs. moderacy in response styles, negative affectivity bias, and social desirability (42).

Third, we used EMA with reports of behavior and affect multiple times per day for multiple days. These individual data points were then summarized in a contemporaneous network representing the associations between certain variables within the individual. Network models are based on correlation between items and, therefore, variance. Due to inspecting variance, the model necessitates fluctuation of item scores or presence of a “flow,” focusing on potential causal relationships (43, 44). This concept is novel and therefore scarce and varies in its operationalization. In this study, the model focused on associations between the nodes of anxiety or depression with nodes representing concepts to target in the corresponding modules. To the best of the authors' knowledge, this has not been done before in the context of assigning treatment modules.

Finally, although therapists seem to have a preference to include patient choice in treatment (45), their input is not often explicitly involved in the module selection process, or, when it is, with less guidance on how to do so (37). Perhaps this is because studies examining the association between patient preference and treatment response find positive, mixed, and negative results (18). Shared decision-making, however, decreases dropout through factors, such as transparency and communication, and both therapist and patient confidence in treatment (11). Therefore, shared decision-making based on data-driven advice with clear instructions instead of merely patient preference might improve the module selection process.

In this study, we describe a multimodal data-driven personalization method and discuss the merits and barriers of this approach. We examine to what extent the three steps of data-driven personalization give congruent recommendations for intervention modules compared to one another and the shared decision of the participant with the expert by experience. First, we hypothesize that the different sources of data-driven personalization advice complement one another rather than replicate. Second, we hypothesize that all data-driven sources of personalization show more congruence than incongruence with the modules that were chosen *via* shared decision-making.

## 2. Materials and methods

StayFine is a study examining the potential of a personalized app-based relapse prevention intervention for anxiety and depressive disorders in remitted adolescents and young adults. Relapse herein is operationalized as the return of an anxiety or depressive disorder as defined by the DSM-5 (46). Inclusion started in December 2019. More detailed information regarding its aim, screening criteria, and procedure can be found in the protocol article (41). For the current study, all steps and materials relevant to the personalization procedure are described below.

### 2.1. Participants

**Figure 1:**
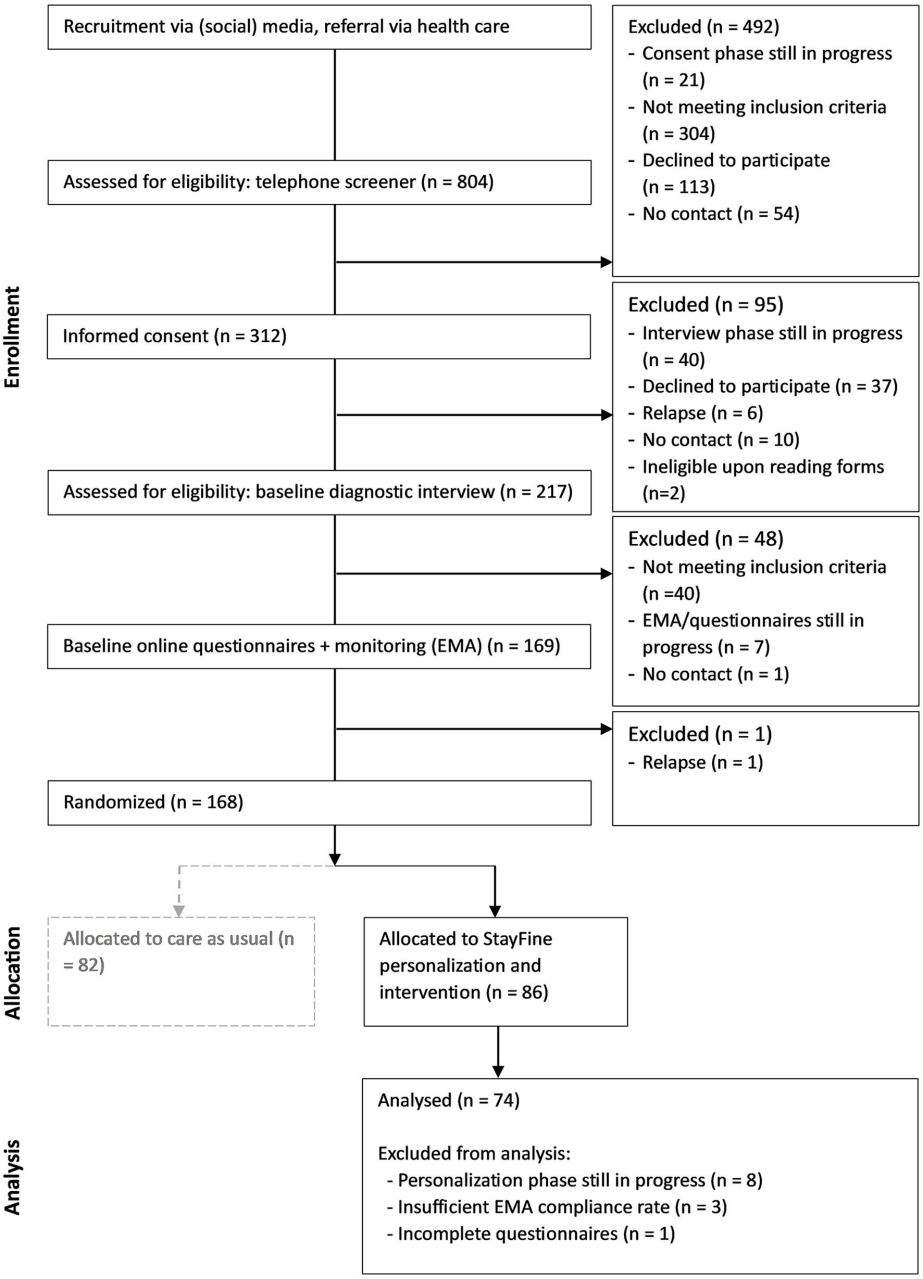
Flow diagram of the study.

A total of 74 participants of the StayFine study who were randomized to the intervention were used in the data analysis of this study as of April 2023. These comprised mostly female individuals (89.19%) with ages ranging from 14 to 21 years (*M* = 19.11; *SD* = 1.78) during the time of the first screening (see Figure 1). All participants had at least one remitted anxiety or depressive disorder, with no current anxiety or depressive disorder for at least 60 days (*M* = 14.90 months, SD = 13.87 months), as measured with the Kiddie Schedule for Affective Disorders and Schizophrenia-lifetime version (K-SADS-PL-DSM-5) (47, 48). The level of residual anxiety symptoms (*M* = 21.64, *SD* = 9.71) was measured by summing the anxiety subscales (separation anxiety disorder, social phobia, generalized anxiety disorder, panic disorder; total of 31 items) of the RCADS [(49) Dutch translation: (50)]. In the age group 14–18 years, the mean score was 13.42 for the summed 31 items in the large epidemiological Dutch study TRAILS, indicating that the anxiety levels were relatively high in our sample (51). The current level of depression (*M* = 5.8, *SD* = 5.01) was in the “none to minimal depression” range [ <10 (52)], resembling an average Dutch student population of 14–20-year-olds [*M* = 5.39, *SD* = 4.81 (53)], as measured with the BDI [(54) Dutch translation: (55)]. For a complete flow of this study (see Figure 1).

The inclusion criteria were Dutch-speaking 13–21-year-olds in remission of a depressive or anxiety disorder, with no current anxiety or depressive disorders, no current alcohol or drug misuse, and no current or prior bipolar disorder (hypo)mania or psychotic episode(s) as measured with the K-SADS-PL-DSM-5 (47). Finally, at entry into the study, participants were excluded if they received psychological treatment more than twice a month, in line with several other relapse prevention studies (56–60). Descriptive of the participants included in this study and their mean values on the questionnaires are depicted in Tables 1, 2.

**Table 1:** Descriptive statistics of the StayFine participants.

**Descriptive**	***n***	**(%)**
**Sex**		
Female	66	(89.19)
Male	8	(10.81)
**Prior disorders** ^a^		
Depressive disorders only	20	(27.03)
Major depressive disorder	20	
Persistent depressive disorder	1	
Anxiety disorders only	7	(9.45)
Agoraphobia	3	
Generalized anxiety disorder	4	
Panic disorder	6	
Separation anxiety disorder	1	
Social anxiety disorder	2	
Specific phobia	0	
Anxiety and depressive disorders	47	(63.51)
Major depressive disorder	45	
Persistent depressive disorder	5	
Agoraphobia	10	
Generalized anxiety disorder	29	
Panic disorder	20	
Separation anxiety disorder	4	
Social anxiety disorder	24	
Specific phobia	18	
**Prior treatment**		
Had prior treatment	62	(83.78)

^a^As measured with the K-SADS-PL-DSM-5.

**Table 2:** Descriptive statistics of the personalization questionnaire totals.

**Descriptive**	***M***	**SD**
**PANAS (affect)**		
Positive affect	33.32	5.19
Negative affect	18.54	6.00
**MHC-SF**^a^ **(flourishing)**			**47.96**	**10.61**
Questions 1–3	11.49	2.20
Questions 4–14	36.47	8.98
**SRSQ (sleep reduction)**			**15.76**	**3.34**

^a^MHC-SF (flourishing) scores are based on values of question 1–3 and 4–14 separately.

### 2.2. Measures

Several means of personalization were used in the StayFine study, which in turn made use of several instruments, as described below.

#### 2.2.1. Clinical diagnostic interview to measure past anxiety and depressive disorders

The K-SADS-PL DSM-5 (47) is a semi-structured diagnostic interview conducted to map prior and current anxiety and depressive disorders in youth according to the DSM-5 (46). A Dutch translation of an online version was administered to participants *via* video call during the baseline assessment of the study by a trained researcher or research assistant with at least a bachelor's degree in psychology or similar. The online version of the K-SADS-PL-DSM-5 has good convergent and instrument validity, with eight assessors showing promising interrater reliability on the screening items of two mock interviews [94 and 96% identical scoring (61)]. For the personalization procedure, the K-SADS-PL-DSM-5 was used to establish the presence of past anxiety and depressive disorders.

#### 2.2.2. Questionnaires to measure sleep quality, affect, and wellness

Several questionnaires are used during the personalization process, conducted through online self-report during the baseline assessments.

To measure sleep, a Dutch version of the *Sleep Reduction Screening Questionnaire* (SRSQ) (62), a shortened version of the *Chronic Sleep Reduction Questionnaire* (63), was used. A higher (total) reduction score indicates lower sleep quality, measured over the past 2 weeks. The SRSQ is a 9-item list with several 3-point scale answers (such as “no,” “sometimes,” and “yes”). It has good internal consistency (α = 0.79) and test-retest reliability in adolescents, with a clinical cutoff value of >17.3 compared to healthy populations (62). In the current study, the same cutoff value was used as an indicator of suboptimal sleep quality.

The positive and negative affect schedule (PANAS) (64) was used to measure affect in the past 2 weeks (65). It is comprised of two 10 per-item alternating scales that measure positive (PA) and negative affect (NA) on a 5-point scale (ranging from “very slightly or not at all” to “extremely”). The Dutch version has good internal consistency (αNA = 0.83, αPA = 0.79; 66). In the current study, cutoff values to determine suboptimal affect were scores lower than 30 for the PA subscale and higher than 30 for the NA subscale, based on averaging the minimum (10) and maximum (49) scores per subscale.

The mental health continuum-short form (MHC-SF) is a 14-item questionnaire that measures emotional (3 items), psychological (6 items), and social (5 items) wellbeing over the past month on a 6-point scale (ranging from “never” to “every day”) (66). In StayFine, the Dutch version by Lamers et al. (67) was used, which was shown to have good internal reliability for the subscales (α =0.74–0.83) and total score (α = 0.89), with moderate test–retest reliability. For flourishing, MHC-SF items 1–3 should include at least one score of 4 or 5, and on items 4–14 more than 6, to indicate healthy flourishing (66). As the cutoff value for advising the module *Wellness* in this sample was incorrectly different from the sub-flourishing threshold for a large subset of the sample, the hypothetical correct advice is shown in the descriptive but excluded from the analyses.

#### 2.2.3. Ecological momentary assessment to measure affect and behavior

**Figure 2:**
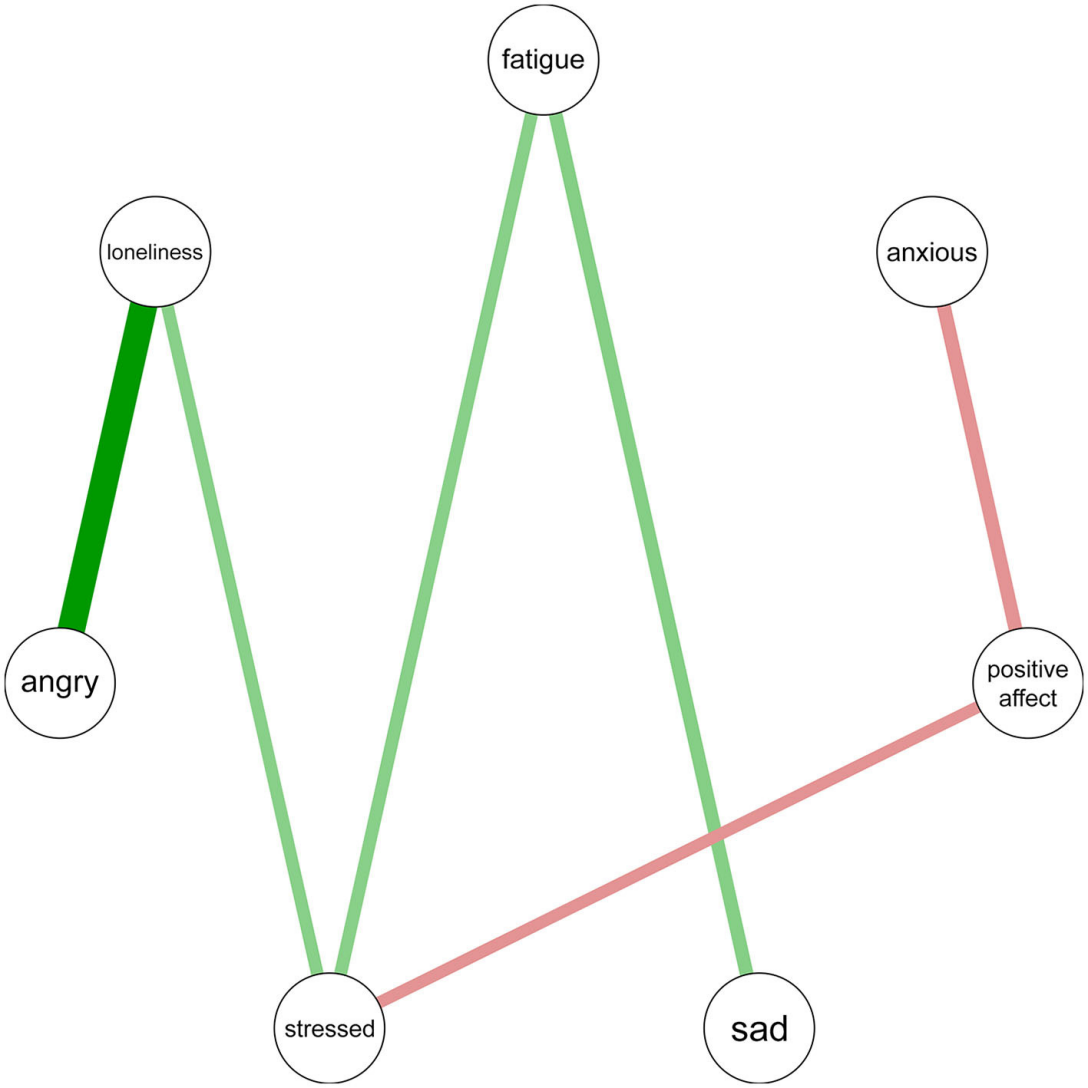
Network model example of the StayFine study, reflecting the past 2 weeks.

The StayFine monitoring is an EMA using a 16-item questionnaire regarding affect and behavior made available through the StayFine app (68), depicted in Supplementary material A. Notifications and optionally alarm clocks were used as reminders to conduct the questionnaire six times a day for 2 weeks. In all, 14 items (e.g., “I feel anxious”) were answered on a 0–100 slider scale. One item (“Were you just with someone else?”) was answered with “yes” or “no.” The last screen has an open text field for comments. Using the packages *qgraph* (v1.9.4) (69), *varhandle* (v2.0.5) (70), *reshape2* (v1.4.4) (71), and *dplyr* (v1.1.1) (72) in R (v4.2.2) (73), this resulted in individual contemporaneous partial correlation symptom networks with 11 nodes: anxious, sad, angry, stressed, positive affect, fatigue, experiential avoidance, behavioral avoidance, loneliness, activity investment, and social company. These networks estimate partial correlations after removing variables with standard deviations lower than 10 and excluding partial correlations smaller than 0.3, similar to the 0.25 of Dobson et al. (74) and others (75, 76). As there is no clear consensus on a required EMA compliance rate in prior research (77, 78), a requirement of ~50 out of 84 EMA measurements was adhered to as the minimum for creating a network (79, 80). The network was visually shared with the expert by experience, together with a small script regarding what modules were recommended based on which connections, to read to the participant (see Figure 2).

### 2.3. Intervention

The StayFine Guided App-Based Personalized Intervention Modules are based on preventive cognitive therapy and CBT ingredients adapted for relapse prevention and consist of psycho-education, diaries, and assignments. Together, they create an individual 10–13-week app-based intervention designed to reduce symptoms and prevent relapse of anxiety and depressive disorders in adolescents and young adults. Each module consists of psycho-education, assignments with personalized automatized feedback, and an optional diary. Guidance, monitoring, and feedback are given *via* the app's chat service by trained experts with experience. Progression to subsequent parts of modules was only possible after this feedback. Experts by experience are individuals who have experienced depressive or anxiety disorders and completed at least a post-secondary vocational education in which they were trained to utilize their experience with mental health problems to help others. A total of six experts by experience were involved in the study, of whom some contributed to the design of the intervention and research, including a review of the personalization method and editing of the personalization script.

Each intervention starts with the fixed modules: *Psycho-education*, including information on relapse (81), introduction of the expert by experience, and instructions regarding the intervention. *Cognitive restructuring* follows, in which rigid dysfunctional attitudes and schemas are identified and re-evaluated through the identification of wishful beliefs that activate positive affect using phantasy and imagination techniques (56, 57, 82, 83). Afterward, three out of five optional modules are followed based on the personalization process. In *Enhancing Positive Affect*, autobiographical memories of positive affect and memories are enhanced using a positive diary and affect labeling, practicing detailed descriptions and positive experiences (56, 57, 82). In *Behavioral Activation*, psycho-education is given on activating oneself to undertake simple pleasurable activities, challenging one to do them, and evaluating the influence on mood afterward. In *Exposure*, psycho-education on anxiety and avoidance is given, and one is challenged to practice exposure and challenge anxious beliefs in various circumstances. In *Sleep*, psycho-education on sleep is given, together with behavioral, cognitive, and relaxation exercises and tips to improve sleep quality. In *Wellness*, psycho-education and exercises are given to improve upon its different dimensions (84). The intervention ends with the last fixed *StayFine plan* module, in which a relapse prevention plan based on prior modules and previous experiences is created.

More details of the intervention and modules are given in the supplementary material of the protocol article (41).

### 2.4. Procedure

#### 2.4.1. Pre-personalization procedure

Participant recruitment took place *via* (social) media, websites, patient organizations, national mental health platforms, schools, and colleges. Interested individuals were contacted by phone for a short screening and verbal information regarding the research. Upon eligibility, they received written information and an informed consent for them to sign (including parents if <16 years) within or after a 2-week period. Upon signing, the diagnostic interview (K-SADS-PL-DSM-5) was performed *via* video call. Upon still meeting inclusion criteria, online questionnaires were sent *via* e-mail to fill out using Castor Electronic Data Capture (85). Simultaneously, a phone call was scheduled for instructions regarding the 2-week EMA, performed six times a day. This was conducted *via* the StayFine app, built into the online secure platform MindDistrict (68). Participants were then randomized to the StayFine intervention or the control condition. Only those who were randomized to the StayFine intervention engaged in the personalization procedure.

#### 2.4.2. Personalization procedure

For determining the 3 of 5 optional modules per participant, a multi-modal personalization procedure was applied using four methods: (1) a semi-structural interview, (2) questionnaires, (3) an individual network model based on EMA, and (4) shared decision-making between the participant and an expert by experience. The first three created data-driven advice that the participant could choose to deviate from or adhere to in step 4. Each step is described below.

The semi-structural diagnostic interview (K-SADS-PL-DSM-5) was used to examine the presence of remitted anxiety or depressive disorders. The module *Behavioral Activation* was advised for a remitted depressive disorder, and *Exposure* was advised for a remitted anxiety disorder. If both disorder types had occurred, both modules were included in the data-driven advice.

As a second step, the online self-report questionnaires (SRSQ, PANAS, and MHC-SF) determined whether to advise the modules *Sleep, Enhancing Positive Affect, and Wellness*, respectively, using the aforementioned cutoff values. The measures refer to the last 2 weeks or the past month.

Third, individual symptom networks were created based on StayFine monitoring. Modules were advised based on the strength and significance of the associations between the anxiety or sadness node and different other nodes. These were the nodes: experiential avoidance, behavioral avoidance, and social company for *Exposure*, social company and activity investment for *Behavioral Activation*, positive affect, anger, and loneliness for *Enhancing Positive Affect*, stressed for *Wellness*, and fatigue for *Sleep*. An illustrative example of a network model is depicted in Figure 2. In this particular example, the following was communicated:

- At moments you feel anxious, you also experience fewer positive feelings.
- At moments you feel sad, you also feel more tired.

This lead to advice of the modules *Enhancing Positive Affect* and *Sleep*, because:

- In the module Enhancing Positive Affect, you train detailed remembrance and recollection of positive events, which may render you less anxious. -
- In the module Sleep you learn how sleep affects how you feel, and how to ensure you sleep long and well enough, so that you may be less tired, which may in turn affect your mood positively.

By observing the model, the authors say that the modules *Enhancing Positive Affect* and *Sleep* could be interesting for this individual, because:

- In the module *Enhancing Positive Affect*, an individual trains detailed remembrance and recollection of positive events, which may render them less anxious.
- In the module *Sleep*, an individual learns how sleep affects how they feel, and how to ensure they sleep long and well enough, so that they may be less tired, which may in turn affect their mood positively.

**Figure 3:**
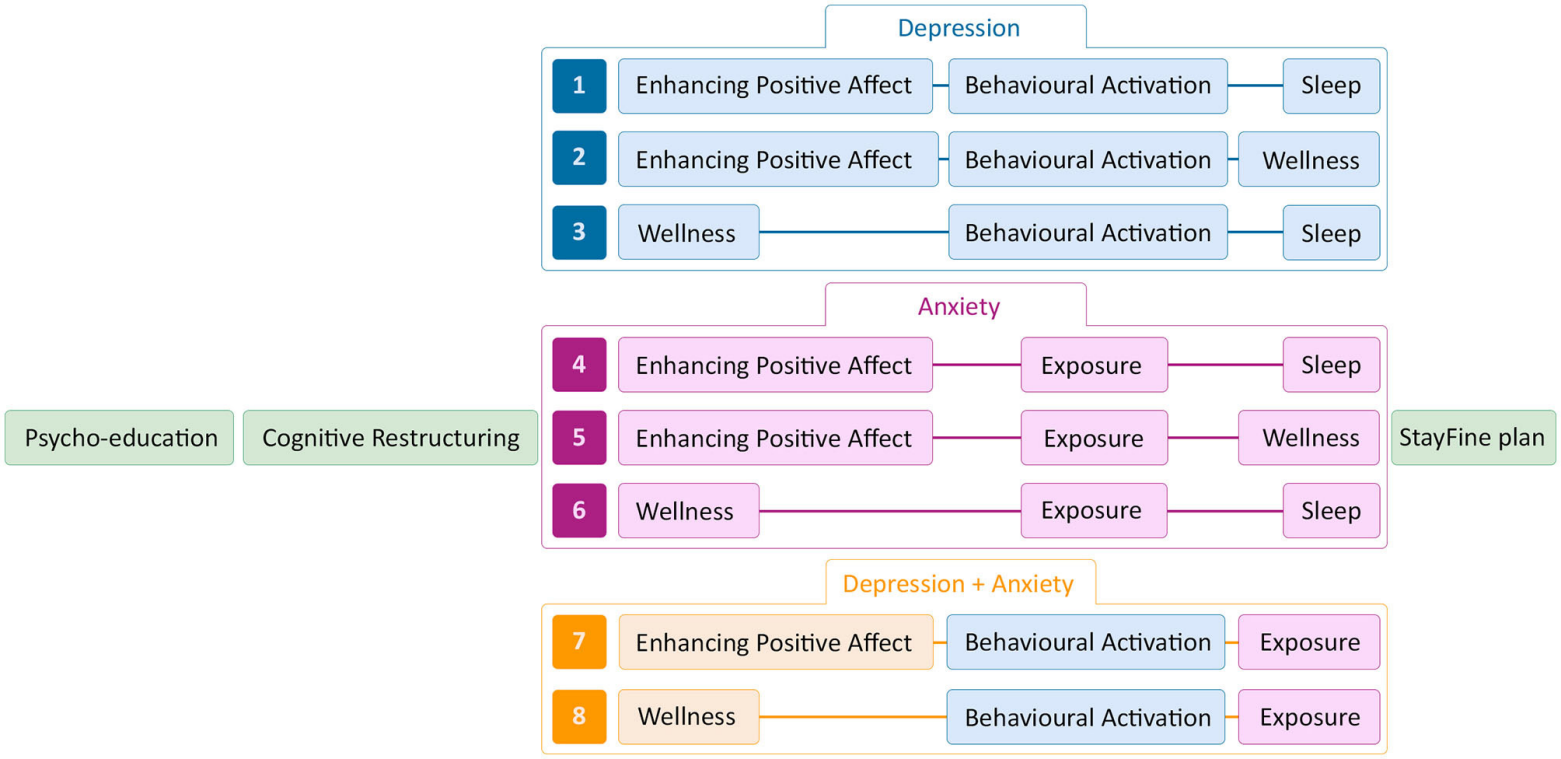
Personalization options of the StayFine study.

Then, through an automated process, the data-driven advice was summarized into a script for the expert by experience to communicate to the participant. This included the recommendation of the corresponding module combinations depicted in Figure 3. If <3 optional modules were advised, all combinations with the highest number of advised modules were recommended (e.g., only advice for *Behavioral Activation* and *Sleep* recommended combinations 1 and 3 of Figure 3). When more than three optional modules were advised, and therefore multiple module combinations were recommended, interview- and questionnaire-based module combinations ranked above combinations including network-based advice. For instance, if *Behavioral Activation, Wellness*, and *Sleep* were advised based on the questionnaires and *Enhancing Positive Affect* based on the network model, module combination 3 in Figure 3 was shown as the first recommendation and options 1 and 2 as the second.

Finally, the participant decided with the expert by experience through shared decision-making which three optional modules to choose in which order. Deviations from recommended module combinations were possible as they were a form of guidance, not a rule, in line with prior modular therapy protocols (37). As can be seen from the figure, *Behavioral Activation* or *Exposure*, or their combination, was always advised to the participant as the main components of depression and anxiety relapse treatment, respectively. Since both modules were extensive, when a combination was advised, the participant was cautioned that options 7 and 8 were more time-consuming.

After modules and their order were chosen, they were delivered to the participant by adding them in order to the participant in the StayFine app. Similar to an online standardized treatment plan, the expert by experience could then view answers to assignments and diaries in the app *via* smartphone or online and give and discuss personalized feedback. Their comparison falls outside the scope of this study, however.

### 2.5. Data analysis

For the data analysis, SPSS (v28) (86) was used to prepare data, gather descriptive information, and calculate frequencies and congruence statistics. First, it was calculated how many participants had to choose options with more or less modules than the interview, questionnaire, and network indicated, as every participant had to choose three modules. Subsequently, frequency tables of advised and chosen modules per personalization step were created. Finally, two contingency tables were made for (1) congruency between the data-driven advice sources and (2) how many participants got a module advised vs. what modules they chose. For the first contingency table, Pearson's chi-square analyses with Yates' continuity correction were performed to examine the association between the data-driven indications.

## 3. Results

All data-driven personalization steps combined, *Exposure* was recommended to 62 (83.78%) participants, *Behavioral Activation* to 67 (90.54%) participants, *Enhancing Positive Affect* to 48 (64.86%) participants, and *Sleep* to 28 (37.84%) participants. *Wellness* was recommended to 63 (85.13%) participants, while it should have been 44 (59.46%) participants. As each participant had to choose 3 of 5 optional modules, 11 (14.86%) participants were recommended <3 modules and 43 (58.11%) more, based on the data-driven advice. The frequencies of advised modules based on diagnostic interviews, questionnaires, and network model indications, and chosen modules through decision-making, are shown in Table 3.

**Table 3:** Frequency table of advised and chosen modules.

**Modules**	**Diagnostic interview Advised**	**Questionnaires Advised**	**Network model Advised**	**Shared decision-making Chosen**
Behavioral Activation	67 (90.54%)	-	4 (5.41%)	52 (70.27%)
Exposure	54 (72.97%)	-	18 (24.32%)	39 (52.70%)
Enhancing positive affect	-	19 (25.68%)	42 (56.75%)	53 (71.62%)
Sleep	-	23 (31.08%)	6 (8.10%)	22 (29.72%)
Wellness	-	33 (44.59%)^a^	19 (25.67%)	56 (75.67%)

^a^Based on if the participant did not flourish.

The frequencies of data-driven advice vs. chosen modules (and disregarding wellness) seem fairly well distributed based on the diagnostic interview and questionnaires. The network model, however, seems to have had a stronger inclination toward *Enhancing Positive Affect* and a disinclination toward *Sleep* and *Behavioral Activation*. Regarding shared decision-making, primary *Sleep*, but also to some degree *Exposure*, seems to have been picked less and *Enhancing Positive Affect* more.

The congruency between the sources for the data-driven advice—the clinical interview and questionnaires vs. the individual symptom network—is depicted in Table 4.

**Table 4:** Contingency table of congruence between advices of different measurement modes.

			**Network model**	
			**No**	**Yes**
Diagnostic interview	Behavioral activation	No	7 (9.45%)	0 (0%)
		Yes	63 (85.14%)	4 (5.41%)
	Exposure	No	12 (16.22%)	8 (10.81%)
		Yes	44 (59.46%)	10 (13.51%)
Questionnaires	Enhancing positive affect	No	26 (35.14%)	29 (39.19%)
		Yes	6 (8.11%)	13 (17.57%)
	Sleep	No	46 (62.16%)	5 (6.76%)
		Yes	22 (29.73%)	1 (1.35%)
	Wellness	No	30 (40.54%)^a^	11 (14.86%)^a^
		Yes	25 (33.78%)^a^	8 (10.81%)^a^

^a^Based on if the participant did not flourish.

Comparing the diagnostic interview- and questionnaire-based advice with the network model-based advice (and disregarding wellness), for *Behavioral Activation*, no chi-square test was performed since one cell has a count of 0. The frequencies show, however, that the network model and diagnostic interview do not give congruent advice regarding whether to do the module. For *Exposure*, the association between the clinical interview and the network model was insignificant [χ^2^(1) = 2.584, *p* = 0.11], meaning the advice between the sources is not significantly associated with one another. Regarding the questionnaires compared to the network model, *Enhancing Positive Affect* [χ^2^(1) = 0.850, *p* = 0.36] was insignificant as well, indicating that the advice is probably not congruent. No chi-square test was performed on *Sleep* advice, comparing questionnaires to the network model, as one cell has a count of 1, but frequencies show the module to be mostly not recommended but also much more often recommended by the questionnaire than the network model. Due to low cell counts, the results of the chi-square tests have to be interpreted with some caution.

Contingency tables of chosen vs. data-driven advised modules are depicted in Table 5.

**Table 5:** Contingency table of advised modules.

		**Diagnostic interview advised**		**Questionnaires advised**		**Network model advised**	
**Modules chosen**		**No**	**Yes**	**No**	**Yes**	**No**	**Yes**
Behavioral Activation	No	6 (8.11%)	16 (21.62%)	-	-	21 (28.38%)	1 (1.35%)
	Yes	1 (1.35%)	51 (68.92%)	-	-	49 (66.22%)	3 (4.05%)
Exposure	No	14 (18.92%)	21 (28.38%)	-	-	29 (39.19%)	6 (8.11%)
	Yes	6 (8.11%)	33 (44.59%)	-	-	27 (36.49%)	12 (16.22%)
Enhancing positive affect	No	-	-	19 (25.68%)	2 (2.70%)	14 (18.92%)	7 (9.46%)
	Yes	-	-	36 (48.65%)	17 (22.97%)	18 (24.32%)	35 (47.30%)
Sleep	No	-	-	46 (62.16%)	6 (8.11%)	49 (66.22%)	3 (4.05%)
	Yes	-	-	5 (6.76%)	17 (22.97%)	19 (25.68%)	3 (4.05%)
Wellness	No	-	-	5 (6.76%)^a^	13 (17.57%)^a^	15 (20.27%)	3 (4.05%)
	Yes	-	-	36 (48.65%)^a^	20 (27.03%)^a^	40 (54.05%)	16 (21.62%)

^a^Based on if the participant did not flourish.

It should be kept in mind that, by definition, congruency goes down after each personalization step since recommended module combinations are somewhat hierarchical. Based on the contingency table (and disregarding wellness), *Behavioral Activation* was chosen congruent with the data-driven advice based on the diagnostic interview. *Exposure* was fairly but less congruent, with a more even spread between cells and the module being more often not chosen despite the advice. Regarding the questionnaires, *Enhancing Positive Affect* seems to have been chosen more often than recommended, incongruent with the data-driven advice. *Sleep* was either picked or more likely not picked congruent with the data-driven advice. Regarding the network models, *Behavioral Activation* seems to have been chosen more than recommended, and *Exposure* as often as not is incongruent with the advice. *Enhancing Positive Affect* does seem to have been chosen more congruently with the given advice, although it was also often chosen even though the network model did not give any inclination to do so. Finally, *Sleep* seems to have been rarely advised based on the network model and more often disregarded than not.

## 4. Discussion

The limitations of current standardized EBTs have given rise to a recent surge of attention to treatment personalization. Modular interventions seem to strike an optimal balance between applying efficacious EBT ingredients while tailoring the intervention to the individual profile of a patient. One point to further examine herein is the integration of data-driven advice and decision-making during module selection, particularly when advice is multimodal. This study describes and critically evaluates the multi-modal data-driven personalization approach of StayFine, additionally examining the extent of congruency between the three data-driven methods and shared decision-making.

First, we examined the frequencies of advised and chosen modules independently. These show that the diagnostic interview—and to a lesser degree the questionnaires—recommended different modules at a fairly similar frequency. This was not the case for the network model and shared decision-making. The network model seemed to favor *Enhancing Positive Affect* and not *Sleep* and *Behavioral Activation*. Regarding shared decision-making, *Enhancing Positive Affect* seemed to be picked more than advised. The overall variance in data-driven advice and decision-making preferences shows the potential benefit of personalization methods combining the two. For instance, a person with sleep problems based on data could be persuaded to reconsider the module despite their initial personal reluctance, thereby improving efficacy. Another person may not need to improve sleeping habits according to available data and therefore does not need to engage in a module that he or she is reluctant about, potentially improving the attrition rate of the intervention.

Second, our hypothesis was that the different sources of data-driven personalization advice complement one another rather than replicate. Thus, we examined the congruence of the advice of the diagnostic interview and questionnaires vs. the individual symptom network models. We found no evidence pointing toward congruency. Questionnaires are prone to capture stable outcomes over a longer time period, and network analyses are better suited to capture fluctuating concepts, so this lack of consistency is not necessarily surprising. Some variables in the network models may have been more likely to have significant associations with daily anxiety and sadness than others, since they have larger fluctuations over the day. For instance, affect is known to have daily fluctuations linked to mental health (87), while fatigue may fluctuate less during daytime. Therefore, the module *Enhancing Positive Affect* may be recommended more by the network model and the module *Sleep* by the questionnaires. Regarding *Behavioral Activation*, it seems plausible that a history of depression, as assessed with a clinical interview, is not *per se* equal to fluctuations of activity during remission, making the advice inherently complementary. Given the incongruency between sources of data-driven advice, this shows clear guidance and methods for incorporation with one another, and decision-making seems warranted.

Finally, we hypothesized that data-driven module recommendations show more congruence than incongruence with the chosen modules *via* shared decision-making. Results showed a preference in the decision-making process. The diagnostic interview was fairly congruent, considering *Exposure* and *Behavioral Activation* are often pitted against one another in the personalization options (see Figure 3), and 58.11% of participants had to pick their optional modules from a larger advised list. The *Exposure* module was likely the first one to be dismissed. Based on the questionnaires, the advice was congruent with the chosen modules regarding *Sleep* but less so with *Enhancing Positive Affect*, which was picked more often. This could be due to *Enhancing Positive Affect*, which focuses on positive thoughts and feelings, whereas low sleep quality and low *Behavioral Activation* are problems that need improvement and may therefore sound less attractive. Perhaps this is also why S*leep—*and to a lesser degree *Exposure*—was picked less than advised. Especially considering adolescents often sleep very less (88) and will more often experience events that disturb good sleep quality, such as parties, which can create a reluctance to improve upon it. Regarding network-based advice, the results seem notably incongruent with the chosen modules. *Behavioral Activation* was chosen more often, *Exposure* as often as not, *Sleep* was rarely advised and chosen even less, and *Enhancing Positive Affect* was chosen congruent with the advice but also often chosen when not recommended. This could have been due to network model-based advice being ranked last in the data-driven advice, therefore being least likely to affect the chosen modules, considering their incongruency with the other data-driven sources.

This study shows that, similar to RP-CBT (89), the development and use of a personalized modular relapse prevention intervention is feasible. Data-driven advice as a basis for shared decision-making could prove a possible improvement (38). Note that the current study focused on the feasibility of data-driven personalization procedures and did not examine the potential benefits in terms of treatment outcome or dropout. Future studies should further examine the efficacy and dropout of personalized modular therapy using data-driven advice compared to personalized modular therapy based on clinician decision-making only and standardized treatment. The benefits should outweigh the costs and efforts of setting up a personalization procedure, including the patient burden of filling out questionnaires, the development of an algorithm, and the training of professionals in discussing date-driven advice in shared decision-making. Different modes of personalization giving incongruent advice on the same abstract concepts tentatively support the idea that different psychometric properties of measurements might capture said abstract concept better, which could increase the robustness of the characteristic (31, 42–44). Yet again, more research is needed with efficacy comparisons to control groups before giving any conclusive results.

There are several limitations to this research. First, due to error, the *Wellness* module was not advised to participants based on whether they flourished or not, thereby advising the module more often than was correct (*n* = 41 incorrectly getting the advice, *n* = 11 incorrectly not getting the advice, and *n* = 22 correctly getting the advice). This does not necessarily detract from the intervention's efficacy, as RP-CBT always includes a wellness module (89), and the intervention combined with medication management outperforms medication management on its own (31, 34). This study is about data-driven personalization; however, it could have influenced choosing the *Wellness* module, and it was therefore excluded from analyses. Second, regarding the shared decision-making process, experts by experience were trained during their education and in StayFine to use their own experience as a source for prompting participants toward their own pathway of sustainable recovery while refraining from limiting recommendations to what worked in their own experience. It cannot, however, be ruled out that they may have had some bias toward their own preferences. Third, it should also be reemphasized that network models necessitate fluctuations in item scores (43, 44). Therefore, whether the item scores of the nodes were high or low in general was not used in the data-driven advice. This makes the assumption that the questionnaire cutoffs compensate for the lack of examining the absolute scores in network models. This is not necessarily the case when there is bad convergent validity, which was not tested. Furthermore, fluctuations of anxiety and sadness nodes could have been influenced by numerous factors, including life events, psychotherapy sessions ( ≤2 per month, *n* = 4), or daily hassles like quarrels with family or friends. It currently remains unexamined whether these factors influence node fluctuations equally. As a fourth point, the questionnaires used in the personalization method only cover three out of the five optional modules. Some other candidate questionnaires could have measured current symptoms of anxiety or levels of (experiential) avoidance and current symptoms of depression or *Behavioral Activation* as indications for advising *Exposure* and *Behavioral Activation*, respectively. Finally, we did not compare our data-driven approach to the commonly used clinical approach of a therapist drawing an individual case formulation in collaboration based on the assessment and narrative of the patient. This is often used for selecting particular intervention modules for an individual. There have also been efforts to combine collaborative case formulations with data-driven approaches [e.g., Burger et al. (90), Sanford et al. (91)]. Further research will progress the field to assess the efficiency and effectiveness of decision tools for selecting modules that are data-driven, therapist-initiated, or combined.

Future research could focus on the addition of within-intervention adjustments to StayFine based on feedback, as is done in MATCH (26) and RP-CBT (89). While data-driven measures with this goal are currently not implemented in StayFine, creating such a feedback system in a relapse prevention intervention could pose an interesting next step in personalizing relapse prevention interventions. One fascinating option herein is personalization of the individual symptom networks through personalization of the EMAs before and during treatment, as illustrated in a case study using Therapy-I involving the therapist and patient (92). In the current study, however, EMA was used with the explicit goal of advising module selection. Another interesting application of EMA, which requires longer continuous measurement than currently done in StayFine, could be the creation of temporaneous individual symptom network models to show when “critical slowing down” occurs. This is when recovery of a system after a disturbance becomes slower, making the critical transition between dynamically stable states possible (93, 94). Early warning signals hereof—increased variance, skewness, temporal autocorrelation, and connectivity—can be used to detect symptom shifts and therefore help as a personalization tool to adjust an intervention for optimal outcomes. It can also give new insights into relapse as a whole, with the network characteristics potentially functioning as a signal for impending relapse (relapse being the re-emergence of symptoms following remission but preceding recovery) (95). Two case studies showed this to be an effective tool in predicting a large increase in depressive symptoms in adults (96, 97). Finally, there is no procedure yet on how to operationalize indicators for recommending modules. One may use questionnaire cutoffs based on questionnaire psychometrics (e.g., the PANAS for affect subscales), more clinical cutoffs (e.g., the SRSQ for sleep reduction), or non-clinical cutoffs (e.g., MHC-SF based on flourishing). Compared to acute treatment, where clinical levels of a disorder seem to be a good indication of starting treatment, there is more uncertainty when it comes to relapse prevention, and each option will provide different recommendations. This also means it cannot be ruled out that the differences between the current data-driven recommendations were to some extent due to the specific thresholds used within. If one changed the questionnaire cutoffs, modules could be recommended more or less often. Without guidelines or procedures to optimally define thresholds and algorithms, efficacy tests with different options can give insights into which indications for which modules are preferable.

In conclusion, it is feasible to personalize a relapse prevention intervention by integrating different complementary data-driven module recommendations into one advice model for shared decision-making. Data-driven sources do seem complementary to one another more than confirmatory, although personalization of *Exposure* and *Behavioral Activation* seemed based mostly on the diagnostic interview, *Sleep* on the questionnaires, and *Enhancing Positive Affect* based on the individual network model. This warrants clear guidelines on the incorporation of data-driven advice within personalized modular interventions. Furthermore, shared decision-making showed a preference for modules improving positive concepts in mental health rather than combating negative ones. This shows multimodal personalization has an additional potential benefit of being able to re-evaluate chosen modules during decision-making to increase efficacy and ignore unnecessary ones to combat attrition rate. However, the efficacy of this benefit has not been examined in the current study. Thus, multimodal personalization in modular interventions is a promising tool and should be prospectively tested for its efficacy.

## Data availability statement

The datasets presented in this article are not readily available because not all participants gave permission for data sharing and this concerns personal sensitive data. Requests to access the datasets should be directed to b.e.a.m.kooiman@rug.nl.

## Ethics statement

The studies involving human participants were reviewed and approved by the Medical-Ethical Review Committee (METC) Utrecht. Written informed consent to participate in this study was provided by the participants' legal guardian(s)/next of kin when participants were below 16 years, and the participants themselves when participants were 16 years or older.

## Author contributions

BK, SR, CA, CB, YS, and MN contributed to conception and design of the study. BK organized the data base, which SR and BK double–checked. BK conducted the analysis, with feedback from CA. BK wrote the first draft of the manuscript, with feedback from MN. All authors contributed to manuscript revision, read, and approved the submitted version.

## References

[1] Kovacs M Obrosky S George C. The course of major depressive disorder from childhood to young adulthood: recovery and recurrence in a longitudinal observational study. J Affect Disord. (2016) 203:374–81. 10.1016/j.jad.2016.05.042 27347807 PMC4975998

[2] Bruce SE Yonkers KA Otto MW Eisen JL Weisberg RB Pagano M . Influence of psychiatric comorbidity on recovery and recurrence in generalized anxiety disorder, social phobia, and panic disorder: a 12-year prospective study. Am J Psychiatry. (2005) 162:1179–87. 10.1176/appi.ajp.162.6.1179 15930067 PMC3272761

[3] Robberegt SJ Brouwer ME Kooiman BE Stikkelbroek YA Nauta MH Bockting CL. Meta-analysis: relapse prevention strategies for depression and anxiety in remitted adolescents and young adults. J Am Acad Child Adol Psychiatr. (2022) 60:306–17. 10.1016/j.jaac.2022.04.014 35513189

[4] Weisz JR Jensen-Doss A Hawley KM. Evidence-based youth psychotherapies versus usual clinical care: a meta-analysis of direct comparisons. Am Psychol. (2006) 61:671. 10.1037/0003-066X.61.7.671 17032068

[5] Thomas R Zimmer-Gembeck MJ Chaffin M. Practitioners' views and use of evidence-based treatment: positive attitudes but missed opportunities in children's services. Admin Policy Mental Health Res. (2014) 41:368–78. 10.1007/s10488-013-0471-y 23371263

[6] Chorpita BF Daleiden EL Weisz JR. Modularity in the Design and Application of Therapeutic Interventions. Applied and Preventive Psychology. (2005) 11:141–56. 10.1016/j.appsy.2005.05.002

[7] James AC Reardon T Soler A James G Creswell C. Cognitive behavioural therapy for anxiety disorders in children and adolescents. Cochrane Datab Syst Rev. (2020) 11:1–5. 10.1002/14651858.CD013162.pub2 33196111 PMC8092480

[8] Curry JF. Future directions in research on psychotherapy for adolescent depression. J Clin Child Adol Psychol. (2014) 43:510–26. 10.1080/15374416.2014.904233 24730421

[9] Ribeiro da Silva D. Personalizing youth psychotherapy: the road ahead. Clin Psychol Sci Pract. (2023) 30:66–9. 10.1037/cps000013424902136

[10] Weisz JR Gray JS. Evidence-based psychotherapy for children and adolescents: data from the present and a model for the future. Child Adolesc Ment Health. (2008) 13:54–65. 10.1111/j.1475-3588.2007.00475.x 32847169

[11] de Soet R Vermeiren R Bansema C van Ewijk H Nijland L Nooteboom L. Drop-out and ineffective treatment in youth with severe and enduring mental health problems: a systematic review. Eur Child Adol Psychiatr. (2023) 54:1–15. 10.1007/s00787-023-02182-z 36882638 PMC11564352

[12] Bennett SD Shafran R. Adaptation, personalization and capacity in mental health treatments: a balancing act? Curr Opin Psychiatry. (2023) 36:28–33. 10.1097/YCO.0000000000000834 36302201 PMC9794160

[13] Ng MY Weisz JR. Annual research review: building a science of personalized intervention for youth mental health. J Child Psychol Psychiatry. (2016) 57:216–36. 10.1111/jcpp.12470 26467325 PMC4760855

[14] Bertie L-A Hudson JL. Cbt for childhood anxiety: reviewing the state of personalised intervention research. Front Psychol. (2021) 12:722546. 10.3389/fpsyg.2021.722546 34899467 PMC8663921

[15] Kraemer HC Wilson GT Fairburn CG Agras WS. Mediators and moderators of treatment effects in randomized clinical trials. Arch Gen Psychiatry. (2002) 59:877–83. 10.1001/archpsyc.59.10.877 12365874

[16] Barber JP Muenz LR. The role of avoidance and obsessiveness in matching patients to cognitive and interpersonal psychotherapy: empirical findings from the treatment for depression collaborative research program. J Consult Clin Psychol. (1996) 64:951. 10.1037/0022-006X.64.5.951 8916624

[17] Beidas RS Lindhiem O Brodman DM Swan A Carper M Cummings C . A probabilistic and individualized approach for predicting treatment gains: an extension and application to anxiety disordered youth. Behav Ther. (2014) 45:126–36. 10.1016/j.beth.2013.05.001 24411120 PMC3893713

[18] Cohen ZD DeRubeis RJ. Treatment selection in depression. Annu Rev Clin Psychol. (2018) 14:209–36. 10.1146/annurev-clinpsy-050817-084746 29494258

[19] Lutz W Rubel JA Schwartz B Schilling V Deisenhofer A-K. Towards integrating personalized feedback research into clinical practice: development of the trier treatment navigator (Ttn). Behav Res Ther. (2019) 120:103438. 10.1016/j.brat.2019.103438 31301550

[20] DeRubeis RJ Cohen ZD Forand NR Fournier JC Gelfand LA Lorenzo-Luaces L. The personalized advantage index: translating research on prediction into individualized treatment recommendations. A Demonstration. PloS ONE. (2014) 9:e83875. 10.1371/journal.pone.0083875 24416178 PMC3885521

[21] Huibers MJ Cohen ZD Lemmens LH Arntz A Peeters FP Cuijpers P . Predicting optimal outcomes in cognitive therapy or interpersonal psychotherapy for depressed individuals using the personalized advantage index approach. PLoS ONE. (2015) 10:e0140771. 10.1371/journal.pone.0140771 26554707 PMC4640504

[22] Cuijpers P Reynolds III CF Donker T Li J Andersson G Beekman A. Personalized treatment of adult depression: medication, psychotherapy, or both? A systematic review. Dep Anxiety. (2012) 29:855–64. 10.1002/da.21985 22815247

[23] Norris LA Kendall PC. Moderators of outcome for youth anxiety treatments: current findings and future directions. J Clin Child Adol Psychol. (2021) 50:450–63. 10.1080/15374416.2020.1833337 33140992 PMC8089117

[24] Simon GE Perlis RH. Personalized medicine for depression: can we match patients with treatments? Am J Psychiatry. (2010) 167:1445–55. 10.1176/appi.ajp.2010.09111680 20843873 PMC3723328

[25] Wright AGC Woods WC. Personalized models of psychopathology. Annu Rev Clin Psychol. (2020) 16:49–74. 10.1146/annurev-clinpsy-102419-125032 32070120

[26] Chorpita BF Weisz JR. MATCH-ADTC: Modular Approach to Therapy for Children with Anxiety, Depression, Trauma, or Conduct Problem: PracticeWise (2009).

[27] Chorpita BF Daleiden EL Park AL Ward AM Levy MC Cromley T . Child steps in California: a cluster randomized effectiveness trial comparing modular treatment with community implemented treatment for youth with anxiety, depression, conduct problems, or traumatic stress. J Consult Clin Psychol. (2017) 85:13–25. 10.1037/ccp0000133 27548030

[28] Chorpita BF Weisz JR Daleiden EL Schoenwald SK Palinkas LA Miranda J . Long-term outcomes for the child steps randomized effectiveness trial: a comparison of modular and standard treatment designs with usual care. J Consult Clin Psychol. (2013) 81:999. 10.1037/a0034200 23978169

[29] Weisz JR Chorpita BF Palinkas LA Schoenwald SK Miranda J Bearman SK . Testing standard and modular designs for psychotherapy treating depression, anxiety, and conduct problems in youth: a randomized effectiveness Trial. Arch Gen Psychiatry. (2012) 69:274–82. 10.1001/archgenpsychiatry.2011.147 22065252

[30] Weisz JR Bearman SK Ugueto AM Herren JA Evans SC Cheron DM . Testing robustness of child steps effects with children and adolescents: a randomized controlled effectiveness trial. J Clin Child Adolesc Psychol. (2020) 49:883–96. 10.1080/15374416.2019.1655757 31517543

[31] Kennard BD Emslie GJ Mayes TL Nightingale-Teresi J Nakonezny PA Hughes JL . Cognitive-behavioral therapy to prevent relapse in pediatric responders to pharmacotherapy for major depressive disorder. J Am Acad Child Adol Psychiatry. (2008) 47:1395–404. 10.1097/CHI.0b013e31818914a1 18978634 PMC2826176

[32] Kaufman J Birmaher B Brent D Rao U Flynn C Moreci P . Schedule for affective disorders and schizophrenia for school-age children-present and lifetime version (K-Sads-Pl): initial reliability and validity data. J Am Acad Child Adol Psychiatry. (1997) 36:980–8. 10.1097/00004583-199707000-00021 9204677

[33] Varni JW Rapoff MA Waldron SA Gragg RA Bernstein BH Lindsley CB. Effects of perceived stress on pediatric chronic pain. J Behav Med. (1996) 19:515–28. 10.1007/BF01904901 8970912

[34] Kennard BD Emslie GJ Mayes TL Nakonezny PA Jones JM Foxwell AA . Sequential treatment with fluoxetine and relapse-prevention CBT to improve outcomes in pediatric depression. Am J Psychiatry. (2014) 171:1083–90. 10.1176/appi.ajp.2014.13111460 24935082 PMC4182111

[35] Huibers MJ Lorenzo-Luaces L Cuijpers P Kazantzis N. On the road to personalized psychotherapy: a research agenda based on cognitive behavior therapy for depression. Front Psychiatry. (2021) 11:607508. 10.3389/fpsyt.2020.607508 33488428 PMC7819891

[36] Paul GL. Strategy of outcome research in psychotherapy. J Consult Psychol. (1967) 31:109. 10.1037/h0024436 5342732

[37] Venturo-Conerly KE Reynolds R Clark M Fitzpatrick OM Weisz JR. Personalizing youth psychotherapy: a scoping review of decision-making in modular treatments. Clin Psychol: Sci Pract. (2023) 30:45. 10.1037/cps0000130

[38] Ægisdóttir S White MJ Spengler PM Maugherman AS Anderson LA Cook RS . The meta-analysis of clinical judgment project: fifty-six years of accumulated research on clinical versus statistical prediction. Couns Psychol. (2006) 34:341–82. 10.1177/0011000005285875

[39] Bauer-Staeb C Griffith E Faraway JJ Button KS. Personalised psychotherapy in primary care: evaluation of data-driven treatment allocation to cognitive–behavioural therapy versus counselling for depression. BJPsych Open. (2023) 9:e46. 10.1192/bjo.2022.628 36861260 PMC10044179

[40] Stumpp NE Sauer-Zavala S. Evidence-based strategies for treatment personalization: a review. Cogn Behav Pract. (2022) 29:902–13. 10.1016/j.cbpra.2021.10.004

[41] Robberegt SJ Kooiman BE Albers CJ Nauta MH Bockting C Stikkelbroek Y. personalised app-based relapse prevention of depressive and anxiety disorders in remitted adolescents and young adults: a protocol of the stayfine Rct. BMJ Open. (2022) 12:e058560. 10.1136/bmjopen-2021-058560 36521888 PMC9756181

[42] Razavi T. Self-Report Measures: An Overview of Concerns and Limitations of Questionnaire Use in Occupational Stress Research. Discussion Paper. University of Southampton (2001).

[43] Bringmann LF Elmer T Epskamp S Krause RW Schoch D Wichers M . What do centrality measures measure in psychological networks? J Abnorm Psychol. (2019) 128:892. 10.1037/abn0000446 31318245

[44] Epskamp S van Borkulo CD van der Veen DC Servaas MN Isvoranu A-M Riese H . Personalized network modeling in psychopathology: the importance of contemporaneous and temporal connections. Clin Psychol Sci. (2018) 6:416–27. 10.1177/2167702617744325 29805918 PMC5952299

[45] Gyani A Shafran R Rose S Lee MJ A. Qualitative investigation of therapists' attitudes towards research: horses for courses? Behav Cogn Psychother. (2015) 43:436–48. 10.1017/S1352465813001069 24330979

[46] American Psychiatric Association. Diagnostic and Statistical Manual of Mental Disorders. Washington DC: American Psychiatric Association Publishing (2013).

[47] Kaufman J Birmaher B Axelson D Perepletchikova F Brent D Ryan N. KSADS-PL-DSM-5. Pittsburgh: Western Psychiatric Institute and Clinic. (2016).

[48] Bockting C. Preventieve Cognitieve Training Bij Terugkerende Depressie. Houten: Bohn Stafleu van Loghum (2009).

[49] Chorpita BF Yim L Moffitt C Umemoto LA Francis SE. Assessment of symptoms of DSM-iv anxiety and depression in children: a revised child anxiety and depression scale. Behav Res Ther. (2000) 38:835–55. 10.1016/S0005-7967(99)00130-8 10937431

[50] Oldehinkel A. Nederlandstalige Vertaling Van De Revised Child Anxiety and Depression Scale (RCADS). (2000).

[51] Mathyssek CM Olino TM Hartman CA Ormel J Verhulst FC Van Oort FV. Does the revised child anxiety and depression scale (RCADS) measure anxiety symptoms consistently across adolescence? The trails study. Int J Methods Psychiatr Res. (2013) 22:27–35. 10.1002/mpr.1380 23483654 PMC3801212

[52] Beck AT Steer RA Carbin MG. Psychometric properties of the beck depression inventory: twenty-five years of evaluation. Clin Psychol Rev. (1988) 8:77–100. 10.1016/0272-7358(88)90050-5

[53] Ruiter M. Preventie van depressie bij jongeren: probleemanalyse, ontwikkeling en evaluatie van de cursus' stemmingmakerij: [Sl: sn] (1997).

[54] Beck AT Ward CH Mendelson M Mock J Erbaugh J. An inventory for measuring depression. Arch Gen Psychiatry. (1961) 4:561–71. 10.1001/archpsyc.1961.01710120031004 13688369

[55] Van der Does A. Handleiding Bij De Nederlandse Versie Van De Beck Depression Inventory [Manual of the Dutch Version of the Bdi]. Lisse, NL: Harcourt Test Publishers. (2002).

[56] Bockting CL Schene AH Spinhoven P Koeter MW Wouters LF Huyser J . Preventing relapse/recurrence in recurrent depression with cognitive therapy: a randomized controlled trial. J Consult Clin Psychol. (2005) 73:647–57. 10.1037/0022-006X.73.4.647 16173852

[57] Bockting CL Klein NS Elgersma HJ van Rijsbergen GD Slofstra C Ormel J . Effectiveness of preventive cognitive therapy while tapering antidepressants versus maintenance antidepressant treatment versus their combination in prevention of depressive relapse or recurrence (DRD study): a three-group, multicentre, randomised controlled trial. The Lancet Psychiatry. (2018) 5:401–10. 10.1016/S2215-0366(18)30100-7 29625762

[58] Kok G Burger H Riper H Cuijpers P Dekker J Van Marwijk H . The three-month effect of mobile internet-based cognitive therapy on the course of depressive symptoms in remitted recurrently depressed patients: results of a randomized controlled trial. Psychother Psychosom. (2015) 84:90–9. 10.1159/000369469 25721915

[59] de Jonge M Bockting CL Kikkert MJ van Dijk MK van Schaik DJ Peen J . Preventive cognitive therapy versus care as usual in cognitive behavioral therapy responders: a randomized controlled trial. J Consult Clin Psychol. (2019) 87:521. 10.1037/ccp0000395 31008635

[60] Brouwer ME Molenaar NM Burger H Williams AD Albers CJ Lambregtse-van den Berg MP . Tapering antidepressants while receiving digital preventive cognitive therapy during pregnancy: an experience sampling methodology trial. Front Psychiatry. (2020) 11:574357. 10.3389/fpsyt.2020.574357 33192705 PMC7641921

[61] Townsend L Kobak K Kearney C Milham M Andreotti C Escalera J . Development of three web-based computerized versions of the kiddie schedule for affective disorders and schizophrenia child psychiatric diagnostic interview: preliminary validity data. J Am Acad Child Adol Psychiatry. (2020) 59:309–25. 10.1016/j.jaac.2019.05.009 31108163

[62] van Maanen A Dewald-Kaufmann JF Oort FJ de Bruin EJ Smits MG Short MA . Screening for sleep reduction in adolescents through self-report: development and validation of the sleep reduction screening questionnaire (SRSQ). Child Youth Care Forum. (2014) 43:607–19. 10.1007/s10566-014-9256-z

[63] Meijer AM. Chronic sleep reduction, functioning at school and school achievement in preadolescents. J Sleep Res. (2008) 17:395–405. 10.1111/j.1365-2869.2008.00677.x 19021856

[64] Watson D Clark LA Tellegen A. Development and validation of brief measures of positive and negative affect: the PANAS scales. J Pers Soc Psychol. (1988) 54:1063. 10.1037/0022-3514.54.6.1063 3397865

[65] Peeters F Ponds R Vermeeren M. Affectiviteit en zelfbeoordeling van depressie en angst. Tijdschr Psychiatr. (1996) 38:240–50.

[66] Keyes CL Wissing M Potgieter JP Temane M Kruger A Van Rooy S. Evaluation of the mental health continuum–short form (MHC–SF) in Setswana-speaking South Africans. Clin Psychol Psychother. (2008) 15:181–92. 10.1002/cpp.572 19115439

[67] Lamers SM Westerhof GJ Bohlmeijer ET Ten Klooster PM Keyes CL. Evaluating the psychometric properties of the mental health continuum-short form (MHC-SF). J Clin Psychol. (2011) 67:99–110. 10.1002/jclp.20741 20973032

[68] MindDistrict BV. Minddistrict. Amsterdam: MindDistrict BV. (2023).

[69] Epskamp S Cramer AO Waldorp LJ Schmittmann VD Borsboom D. Qgraph: network visualizations of relationships in psychometric data. J Stat Softw. (2012) 48:1–18. 10.18637/jss.v048.i0427534393

[70] Mahmoudian M. Varhandle: Functions for Robust Variable Handling. R package version 2.0.5 (2020). Available online at: https://CRAN.R-project.org/package=varhandle

[71] Wickham H. Reshaping data with the reshape package. J Stat Softw. (2007) 21:1–20. Available online at: http://www.jstatsoft.org/v21/i12/

[72] Wickham H François R Henry L Müller K Vaughan D. dplyr: A Grammar of Data Manipulation. R package version 1.1.1 (2023). Available online at: https://CRAN.R-project.org/package=dplyr

[73] R Core Team. R: A Language and Environment for Statistical Computing. Vienna, Austria: R Foundation for Statistical Computing (2022).

[74] Dobson ET Croarkin PE Schroeder HK Varney ST Mossman SA Cecil K . Bridging anxiety and depression: a network approach in anxious adolescents. J Affect Disord. (2021) 280:305–14. 10.1016/j.jad.2020.11.027 33221716 PMC7744436

[75] Epskamp S Borsboom D Fried EI. Estimating psychological networks and their accuracy: a tutorial paper. Behav Res Methods. (2018) 50:195–212. 10.3758/s13428-017-0862-1 28342071 PMC5809547

[76] Jones PJ Mair P Riemann BC Mugno BL McNally RJ. A network perspective on comorbid depression in adolescents with obsessive-compulsive disorder. J Anxiety Disord. (2018) 53:1–8. 10.1016/j.janxdis.2017.09.008 29125957

[77] Jaso BA Kraus NI Heller AS. Identification of careless responding in ecological momentary assessment research: from post-hoc analyses to real-time data monitoring. Psychol Methods. (2022) 27:958. 10.1037/met0000312 34582244 PMC11565177

[78] Heininga VE Dejonckheere E Houben M Obbels J Sienaert P Leroy B . The dynamical signature of anhedonia in major depressive disorder: positive emotion dynamics, reactivity, and recovery. BMC Psychiatry. (2019) 19:1–11. 10.1186/s12888-018-1983-5 30736751 PMC6368777

[79] Dejonckheere E Mestdagh M Houben M Rutten I Sels L Kuppens P . Complex affect dynamics add limited information to the prediction of psychological well-being. Nat Hum Behav. (2019) 3:478–91. 10.1038/s41562-019-0555-0 30988484

[80] Bos FM Schoevers RA Aan Het Rot M. Experience sampling and ecological momentary assessment studies in psychopharmacology: a systematic review. Eur Neuropsychopharmacol. (2015) 25:1853–64 10.1016/j.euroneuro.2015.08.008 26336868

[81] Bockting CL. Preventieve Cognitieve Training Bij Terugkerende Depressie. Houten: Bohn Stafleu van Loghum (2009).

[82] Bockting CL Spinhoven P Wouters LF Koeter MW Schene AH. Long-term effects of preventive cognitive therapy in recurrent depression: a 55-year follow-up study. J Clin Psychiatry. (2009) 16:1621. 10.4088/JCP.08m04784blu 20141705

[83] Padesky C. Center for Cognitive Therapy. (2023). Available online at: https://www.padesky.com/ (accessed May 25, 2023).

[84] Ryff CD Singer BH. Know thyself and become what you are: a eudaimonic approach to psychological well-being. J Happiness Stud. (2008) 9:13–39. 10.1007/s10902-006-9019-0

[85] Castor EDC. Castor Electronic Data Capture. Amsterdam: Ciwit BV. (2023).

[86] IBM Corp. Ibm Spss Statistics for Windows. 28.0.1.0 ed. Armonk, NY: IBM Corp (2022).

[87] Reitsema AM Jeronimus BF van Dijk M de Jonge P. Emotion dynamics in children and adolescents: a meta-analytic and descriptive review. Emotion. (2022) 22:374. 10.1037/emo0000970 34843305

[88] Wheaton AG Jones SE Cooper AC Croft JB. Short sleep duration among middle school and high school students—United States, 2015. Morbid Mortal Wkly Rep. (2018) 67:85. 10.15585/mmwr.mm6703a1 29370154 PMC5812312

[89] Kennard BD Stewart SM Hughes JL Jarrett RB Emslie GJ. Developing cognitive behavioral therapy to prevent depressive relapse in youth. Cogn Behav Pract. (2008) 15:387–99. 10.1016/j.cbpra.2008.02.006 20535241 PMC2882305

[90] Burger J Epskamp S van der Veen DC Dablander F Schoevers RA Fried EI . A clinical premise for personalized models: toward a formal integration of case formulations and statistical networks. J Psychopathol Clin Sci. (2022) 131:906. 10.1037/abn0000779 36326631

[91] Sanford BT Ciarrochi J Hofmann SG Chin F Gates KM Hayes SC. Toward empirical process-based case conceptualization: an idionomic network examination of the process-based assessment tool. J Cont Behav Sci. (2022) 25:10–25. 10.1016/j.jcbs.2022.05.006

[92] von Klipstein L Servaas MN Schoevers RA van der Veen DC Riese H. Integrating personalized experience sampling in psychotherapy: a case illustration of the therap-I module. Heliyon. (2023) 9:e14507. 10.1016/j.heliyon.2023.e14507 36967959 PMC10036928

[93] Helmich MA Smit AC Bringmann LF Schreuder MJ Oldehinkel AJ Wichers M . Detecting impending symptom transitions using early-warning signals in individuals receiving treatment for depression. Clin Psychol Sci. (2021) 2:21677026221137006. 10.31234/osf.io/vf86s

[94] Helmich MA Olthof M Oldehinkel AJ Wichers M Bringmann LF Smit AC. Early warning signals and critical transitions in psychopathology: challenges and recommendations. Curr Opin Psychol. (2021) 41:51–8. 10.1016/j.copsyc.2021.02.008 33774486

[95] Bockting CL Hollon SD Jarrett RB Kuyken W Dobson K A. Lifetime approach to major depressive disorder: the contributions of psychological interventions in preventing relapse and recurrence. Clin Psychol Rev. (2015) 41:16–26. 10.1016/j.cpr.2015.02.003 25754289

[96] Wichers M Smit AC Snippe E. Early warning signals based on momentary affect dynamics can expose nearby transitions in depression: a confirmatory single-subject time-series study. J Person-Oriented Res. (2020) 6:1. 10.17505/jpor.2020.22042 33569148 PMC7842626

[97] Wichers M Groot PC Psychosystems E Group E. Critical slowing down as a personalized early warning signal for depression. Psychother Psychosom. (2016) 85:114–6. 10.1159/000441458 26821231

