# Supplementary material for: Congruency of multimodal data-driven personalization with shared decision-making for StayFine: individualized app-based relapse prevention for anxiety and depression in young people
Source: Front Psychiatry. 2023 Sep 29;14:1229713. doi: 10.3389/fpsyt.2023.1229713 (PMC10570515; doi:10.3389/fpsyt.2023.1229713)
Supplement: Supplementary file 1 [file Table_1.DOCX]

Supplementary Material A

Questions asked during the monitoring (EMA) and their respective nodes and module recommendations.

| Question | Scale | Node | Module |
| --- | --- | --- | --- |
| 1. I feel anxious. | 0-100 | Anxious | Exposure |
| 1. I feel energetic. | 0-100 | Positive affect | Enhancing positive affect |
| 1. I feel sad. | 0-100 | Sad | Behavioral activation |
| 1. I feel angry. | 0-100 | Angry | Enhancing positive affect |
| 1. I feel relaxed. | 0-100 | Positive affect | Enhancing positive affect |
| 1. I feel stressed. | 0-100 | Stressed | Wellness |
| 1. I feel enthusiastic. | 0-100 | Positive affect | Enhancing positive affect |
| 1. I feel cheerful. | 0-100 | Positive affect | Enhancing positive affect |
| 1. I feel tired. | 0-100 | Fatigue | Sleep |
| 1. I want to suppress my feelings. | 0-100 | Experiential avoidance | Exposure |
| 1. I avoid difficult things. | 0-100 | Behavioral avoidance | Exposure |
| 1. I feel lonely. | 0-100 | Loneliness | Enhancing positive affect |
| 1. My current activity was enjoyable. | 0-100 | Activity investment | Behavioral activation |
| 1. My current energy costed energy. | 0-100 | Activity investment | Behavioral activation |
| 1. Were you just with someone else? | 0-1 | Social company | Exposure Behavioral activation |
| 1. You can leave a message here. We will read this, but not directly. If you require immediate help, please contact your general practitioner. | Open question | - | - |
